# Granulocyte colony‐stimulating factor‐associated aortitis in a man with advanced prostate cancer

**DOI:** 10.1002/iju5.12835

**Published:** 2025-02-13

**Authors:** Ryota Ikadai, Goshi Kitano, Manabu Kato, Takahiro Kojima

**Affiliations:** ^1^ Department of Urology Aichi Cancer Center Hospital Nagoya Aichi Japan

**Keywords:** aortitis, chemotherapy, fever, granulocyte colony‐stimulating factor, prostate cancer

## Abstract

**Introduction:**

Granulocyte colony‐stimulating factor‐associated aortitis remains poorly understood among clinicians.

**Case presentation:**

We present a case of G‐CSF‐associated aortitis in a 70‐year‐old male with stage IVb castration‐resistant prostate cancer (cT3bN0M1b) receiving docetaxel chemotherapy. Neutropenia (280/μL) developed on day 8 of the first chemotherapy cycle, prompting subcutaneous administration of filgrastim, a short‐acting G‐CSF, on days 8–10. On day 14, the patient presented to the outpatient clinic with fever but no other significant symptoms. Computed tomography revealed filgrastim‐induced thoracic aortitis. Daily prednisone treatment (equivalent to 25 mg prednisolone) was initiated on the following day. Although the initial episode of aortitis resolved within 5 weeks, subsequent pegfilgrastim resulted in recurrence around the left subclavian artery, necessitating further steroid therapy.

**Conclusion:**

Persistent high fever following G‐CSF administration may indicate drug‐induced aortitis, highlighting the potential for aortitis recurrence with repeated G‐CSF use.

**Keynote message**

We encountered a case of aortitis in a patient with advanced prostate cancer receiving docetaxel chemotherapy followed by granulocyte colony‐stimulating factor (G‐CSF) administration. Resumption of G‐CSF should be determined on a case‐by‐case basis. This decision should be made by weighing the risk of aortitis against infection prophylaxis.

**Abbreviations & Acronyms**
BT: body temperature
CRP: C‐reactive protein
CT: computed tomography
G‐CSF: granulocyte colony‐stimulating factor

## Introduction

Myelosuppressive cytotoxic chemotherapy can induce severe neutropenia, increasing the risk of fatal infections, particularly febrile neutropenia.^1^ Recombinant human G‐CSFs, including filgrastim and PEGylated filgrastim (pegfilgrastim), have been extensively studied and widely utilized to mitigate the severity and duration of chemotherapy‐induced neutropenia and associated infectious complications.^1^ While the most common side effects are tolerable, such as bone pains, headache, and fatigue,^2^ G‐CSF‐associated aortitis is a less recognized but potentially serious complication.^3^, ^4^, ^5^ Herein, we report a case of G‐CSF‐associated aortitis in a patient with advanced prostate cancer. This case underscores the importance of proper treatment planning for G‐CSF‐associated aortitis and the underlying malignancies. Furthermore, the recommended chemotherapy regimens for patients with G‐CSF‐associated aortitis are discussed.

## Case presentation

**Fig. 1:**
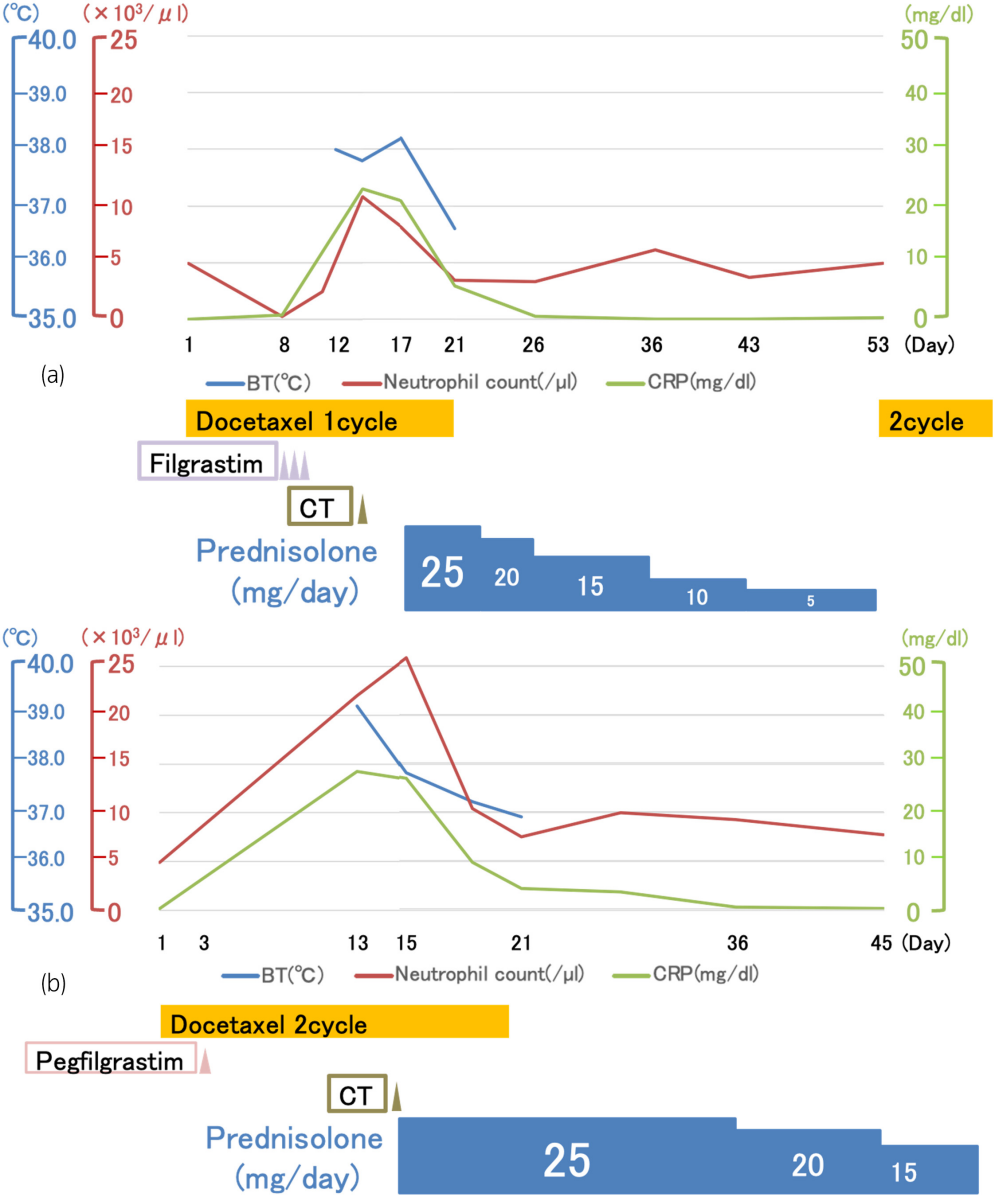
Clinical course. The patient's fever and CRP levels rapidly improved following steroid administration. (a) The first chemotherapy cycle. (b) The second chemotherapy cycle.

**Fig. 2:**
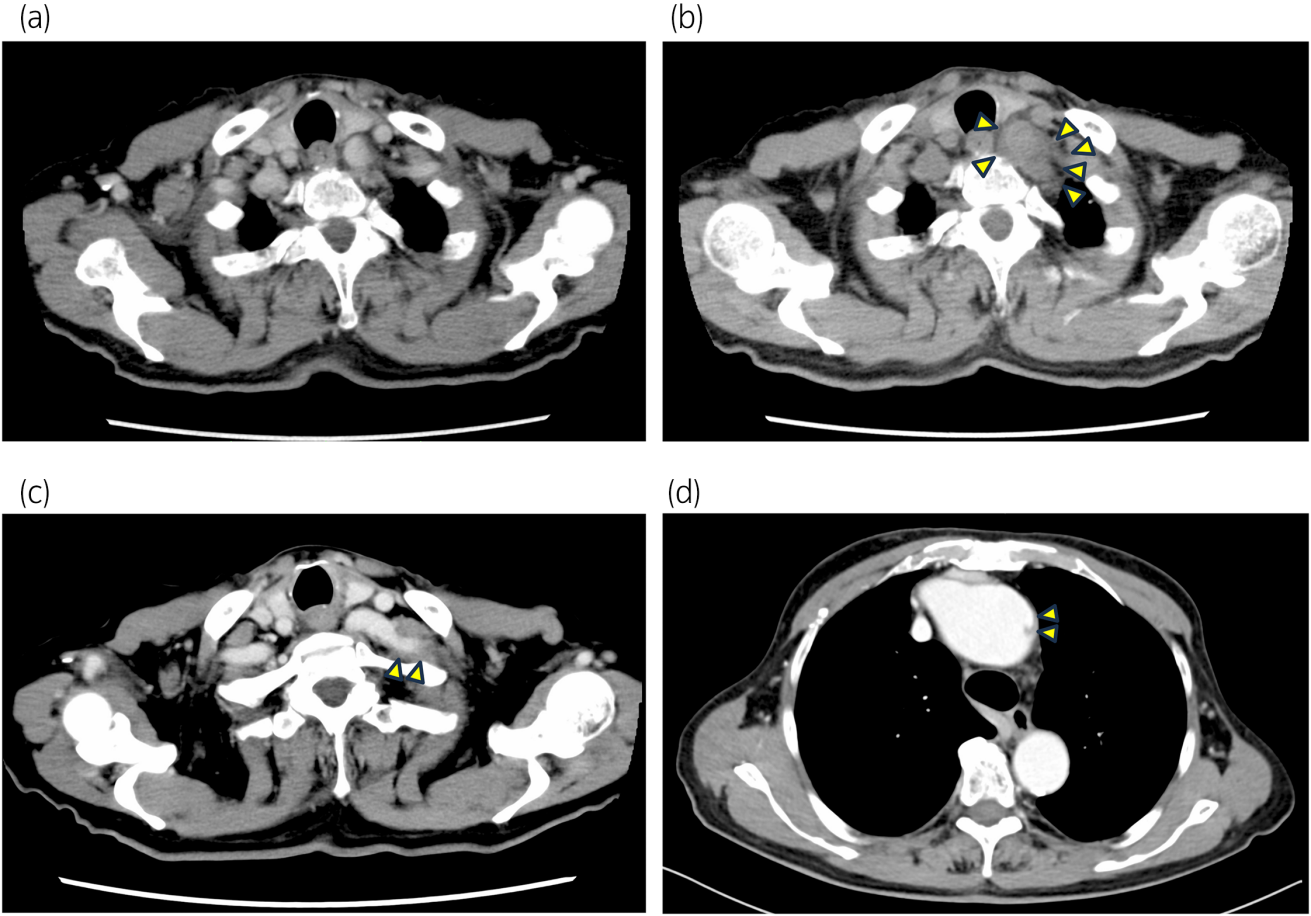
Computed tomography images. (a) Image obtained before the first administration of filgrastim. (b) Aortic arch wall thickening is observed after filgrastim administration. (c) Left subclavian artery wall thickening is reported following pegfilgrastim administration. (d) A small aortic dissection of the aortic arch is noted 6 months after the first chemotherapy cycle.

The patient was a 70‐year‐old male diagnosed with castration‐resistant stage IVb prostate cancer (cT3bN0M1b), and docetaxel chemotherapy was initiated. On day 8 of the first cycle, laboratory tests revealed neutropenia (280/μL). Filgrastim was administered subcutaneously from days 8 to 10, resulting in an improved neutrophil count of 2481/μL on day 11. On day 12, the patient developed intermittent fever (up to 38°C) despite receiving levofloxacin. In the absence of other objective symptoms, he presented to our outpatient clinic on day 14, where work‐up showed leukocytosis (10 823/μL) and elevated C‐reactive protein levels (22.98 mg/dL) (Fig. 1a). Plain computed tomography (CT) demonstrated thickening of the aortic arch wall (Fig. 2b), raising the suspicion for filgrastim‐induced thoracic aortitis. Persistent high fever was observed on day 17. Levofloxacin was discontinued after negative culture results, and daily prednisone (equivalent to 25 mg prednisolone) was initiated. Steroid treatment was gradually tapered over a 5‐week period. Following resolution within 5 weeks, steroid treatment was discontinued, and docetaxel chemotherapy was resumed. Based on the previous course, a different G‐CSF (pegfilgrastim) was started on day 3 of the second cycle. However, on day 15, aortitis recurred around the left subclavian artery, accompanied by leukocytosis (25 855/μL). Daily prednisone was promptly re‐initiated (Figs. 1b and 2c). During follow‐up, CT revealed a small aortic dissection of the aortic arch 6 months after the first chemotherapy cycle (Fig. 2d). The patient's condition was maintained with prednisolone treatment (5 mg daily), anti‐impulse therapy to minimize aortic wall stress, and continued docetaxel chemotherapy. Approximately 6 months later, prostate cancer progression was observed. Additionally, comprehensive genomic profiling revealed microsatellite instability‐high cancer, necessitating pembrolizumab therapy. No progression was observed in this patient for 3 months after starting Pembrolizumab treatment.

## Discussion

While the occurrence of G‐CSF‐associated aortitis has been increasingly recognized, its natural course remains poorly understood. Distinguishing aortitis caused solely by G‐CSF from that due to anti‐cancer therapies is challenging. However, considering the widespread use of pegfilgrastim among patients with cancer, G‐CSF should be considered a potential etiology when aortitis develops.^6^ G‐CSF‐associated aortitis commonly affects those aged over 50 years, with a male predominance (9:1), usually occurring within 10 days of G‐CSF administration.^7^ CT imaging typically reveals aortic wall or periaortic tissue thickening,^6^ with the thoracic or thoracoabdominal aorta being the most involved site.^7^ Given the effectiveness of corticosteroids and tocilizumab (IL‐6 antagonist) for Takayasu and giant cell aortitis, steroids may be considered a reasonable treatment approach for this condition.

Spontaneous resolution without steroids within 2 weeks has been observed in less than half of all aortitis cases, with more than half being reported from Japan.^7^ Specifically, Japanese patients treated with G‐CSF showed an aortitis incidence of 0.47%. Although the incidence appears higher in patients treated with PEGylated G‐CSFs compared to other G‐CSF agents.^5^

To the best of our knowledge, only a limited number of case reports on G‐CSF‐associated aortitis recurrence have been published.^6^, ^8^, ^9^, ^10^, ^11^, ^12^ For these cases, the median age was 62 years (range: 45–76 years), most were female (*n* = 5; 71.4%), and all came from Asian populations (Table 1). Fever was the most common presenting symptom, which was observed in five cases (71.4%), and pegfilgrastim, a long‐acting G‐CSF, was administered six cases (85.7%).^6^, ^8^, ^9^, ^10^, ^11^ Thus, compared to lenograstim and filgrastim, this suggests an association between pegfilgrastim and increased aortitis recurrence. Intense acute inflammation was also reported to rarely cause complications, such as asymptomatic aortic dissection.^13^

**Table 1:** Characteristics of G‐CSF‐associated aortitis recurrence (*n* = 7)

Characteristic	No. (*n* = 7)	%
Reported year	2017–2024	
Age (years)	62 (45–76)*	
Sex		
Male	2	28.6
Female	5	71.4
Race		
Asian	7	100
Primary disease		
Lymphoma	3	42.8
Breast cancer	1	14.3
Ovarian cancer	1	14.3
Pancreatic cancer	1	14.3
Prostate cancer (our case)	1	14.3
G‐CSF (1st → 2nd)		
Pegfilgrastim → Pegfilgrastim	4	57.1
Filgrastim → Pegfilgrastim	1	14.3
Pegfilgrastim → Filgrastim	1	14.3
Lenograstim → Filgrastim	1	14.3
Symptom (with duplication)		
Fever	5	71.4
Pain (back, chest)	2	28.6
Not reported	2	28.6

* The number in parentheses is a range.

One case involved the presence of human leukocyte antigen B52 (HLA‐B52), which is associated with Takayasu arteritis.^12^, ^14^ Moreover, approximately 90% of such cases were female.^15^ This trend is similar to G‐CSF‐associated aortitis, indicating potential shared pathogenic mechanisms. For instance, the involvement of natural killer cells in Takayasu arteritis, as reported in genome‐wide association studies, may also be implicated in G‐CSF‐associated aortitis.^14^

This case underscores that avoidance of all G‐CSF agents should be considered in patients with G‐CSF‐associated aortitis. Deciding on resuming G‐CSF therapy in subsequent cycles should be made on a case‐by‐case basis by weighting the risk of aortitis against the benefits of infection prophylaxis.

## Conclusion

This case report describes a male patient with advanced prostate cancer who experienced recurrent episodes of G‐CSF‐associated aortitis. Due to the risk of serious complications, including asymptomatic aortic dissection, G‐CSF‐associated aortitis should be considered as a potential adverse event prior to administration. In addition, it is important to recognize the risk of recurrence with repeated G‐CSF use.

## Author contributions

Ryota Ikadai: Writing – original draft. Goshi Kitano: Writing – review and editing. Manabu Kato: Writing – review and editing. Takahiro Kojima: Writing – review and editing.

## Conflict of interest

The authors declare no conflict of interest.

## Approval of the research protocol by an Institutional Reviewer Board

Not applicable.

## Informed consent

Written informed consent was obtained from the patient for the publication of this report.

## Registry and the Registration No. of the study/trial

Not applicable.
